# Supplementary material for: Transitive inference in cleaner wrasses (Labroides dimidiatus)
Source: PLoS One. 2020 Aug 18;15(8):e0237817. doi: 10.1371/journal.pone.0237817 (PMC7433877; doi:10.1371/journal.pone.0237817)
Supplement: S1 Fig — (PDF) [file pone.0237817.s001.pdf]

Figure S1

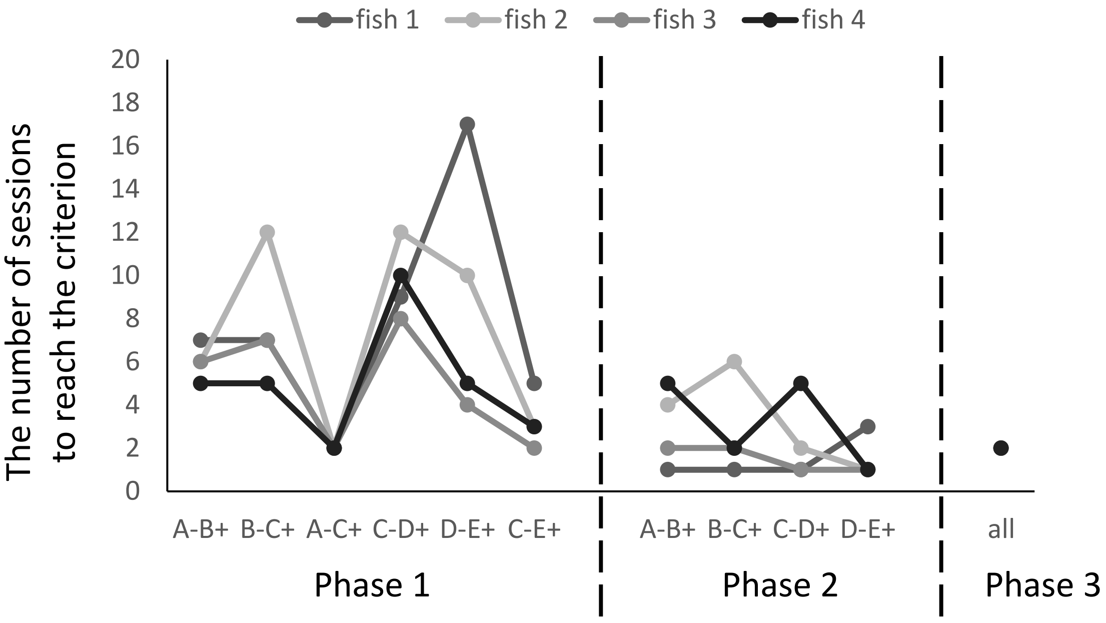

Figure S1. The number of sessions for fulfilling the criteria during the training stages for each subject.
