# Supplementary material for: Transitive inference in cleaner wrasses (Labroides dimidiatus)
Source: PLoS One. 2020 Aug 18;15(8):e0237817. doi: 10.1371/journal.pone.0237817 (PMC7433877; doi:10.1371/journal.pone.0237817)
Supplement: S1 Table — (PDF) [file pone.0237817.s002.pdf]

Table S1 The color type used for each subject

	Color				
ID	A	B	C	D	E
Fish 1	red	white	green	black	blue
Fish 2	red	black	yellow	white	green
Fish 3	blue	black	yellow	white	Red
Fish 4	green	white	red	black	yellow
