# Supplementary material for: Transitive inference in cleaner wrasses (Labroides dimidiatus)
Source: PLoS One. 2020 Aug 18;15(8):e0237817. doi: 10.1371/journal.pone.0237817 (PMC7433877; doi:10.1371/journal.pone.0237817)
Supplement: S2 Table — Asterisk means p < 0.05 (binomial test). (PDF) [file pone.0237817.s003.pdf]

Table S2 The number of correct choices out of total number of adjacent pair trials before BD test and number of choices of D on BD test in test phase. *: $p < 0.05$ (binomial test).

| ID | adjacent pairs | | | | |
|---|---|---|---|---|---|
| | A-B+ | B-C+ | C-D+ | D-E+ | BD |
| fish 1 | 6/6 | 3/6 | 4/6 | 6/6 | 10/12* |
| fish 2 | 5/6 | 5/6 | 5/6 | 4/6 | 10/12* |
| fish 3 | 6/6 | 6/6 | 6/6 | 6/6 | 12/12* |
| fish 4 | 6/6 | 6/6 | 6/6 | 6/6 | 12/12* |
