# Supplementary material for: Transitive inference in cleaner wrasses (Labroides dimidiatus)
Source: PLoS One. 2020 Aug 18;15(8):e0237817. doi: 10.1371/journal.pone.0237817 (PMC7433877; doi:10.1371/journal.pone.0237817)
Supplement: S3 Table — LSD means the least-square difference between the proportion of correct responses predicted by the model and obtained in the training phase. ß, ε, α, γ, A are the model parameters [4]. (PDF) [file pone.0237817.s004.pdf]

Table S3 Each parameters and least-square difference of best fitted models (the original and modified Siemann-Delius and Wynne). LSD means the least-square difference between the proportion of correct responses predicted by the model and obtained in the training phase. ß, ε, α, γ, A are the model parameters (see Lasareva et al. 2004)

	Fish 1	Fish 2	Fish 3	Fish 4
Siemann-Delius				
ß$_{+}$	0.1926	0.0505	0.4486	0.4292
ß$_{-}$	0.3771	0.0000	0.6806	0.5870
Ε	0.4505	0.1883	0.2821	0.1383
LSD	0.1388	0.0207	0.1093	0.0160
Wynne				
A	1.9400	1.8348	33.8773	311.0154
ß	0.2089	0.2881	0.6910	0.5580
Γ	0.4894	0.3872	0.4903	0.5937
LSD	0.0512	0.0305	0.0639	0.2000
Siemann-Delius with value transfer				
ß$_{+}$	0.2502	0.0000	0.2309	0.2993
ß$_{-}$	0.4308	0.0925	0.6440	0.4889
Ε	0.5660	0.4398	0.1663	0.1704
A_{+}	0.0465	0.0120	0.0550	0.0517
A_{-}	0.0282	0.0427	0.0512	0.0479
LSD	0.0070	0.0192	0.0688	0.1064
Wynne with value transfer				
A	6.7664	5.2839	5934.4380	7.6367
ß	0.1175	0.0826	0.4933	0.6985
Γ	1.0000	0.7906	0.4903	0.0492
A_{+}	0.0000	0.0713	0.2493	0.1839
A_{-}	0.0000	0.3908	0.2428	0.2740
LSD	0.0497	0.0172	0.0000	0.0645
