# Supplementary material for: Transitive inference in cleaner wrasses (Labroides dimidiatus)
Source: PLoS One. 2020 Aug 18;15(8):e0237817. doi: 10.1371/journal.pone.0237817 (PMC7433877; doi:10.1371/journal.pone.0237817)
Supplement: S4 Table — (PDF) [file pone.0237817.s005.pdf]

Table S4. Simulated accuracies for the Siemann-Delium and Wynne models

ID		A-B+	B-C+	C-D+	D-E+	BD
Fish 1	Obtained	100.00	50.00	66.67	100.00	83.33
	Siemann-Delius	100.00	50.00	66.67	100.00	0.03
	Wynne	88.80	66.82	80.54	79.04	45.26
Fish 2	Obtained	83.33	83.33	83.33	66.67	83.33
	Siemann-Delius	78.72	88.98	79.36	70.79	52.50
	Wynne	85.08	82.78	73.32	80.53	50.83
Fish 3	Obtained	100.00	100.00	100.00	100.00	100.00
	Siemann-Delius	100.00	100.00	99.98	99.98	26.08
	Wynne	100.00	100.00	100.00	100.00	100.00
Fish 4	Obtained	100.00	100.00	100.00	100.00	100.00
	Siemann-Delius	100.00	100.00	100.00	99.98	71.74
	Wynne	99.37	99.97	99.83	99.93	59.47
