# Supplementary material for: Transitive inference in cleaner wrasses (Labroides dimidiatus)
Source: PLoS One. 2020 Aug 18;15(8):e0237817. doi: 10.1371/journal.pone.0237817 (PMC7433877; doi:10.1371/journal.pone.0237817)
Supplement: S5 Table — (PDF) [file pone.0237817.s006.pdf]

Table S5. Simulated accuracies for the modified Siemann-Delium and Wynne models incorporating a value transfer mechanism

ID		A-B+	B-C+	C-D+	D-E+	BD
Fish 1	Obtained	100.00	50.00	66.67	100.00	83.33
	Siemann-Delius	100.00	51.64	81.40	89.75	88.18
	Wynne	88.80	66.82	80.45	79.04	45.26
Fish 2	Obtained	83.33	83.33	83.33	66.67	83.33
	Siemann-Delius	94.19	84.49	80.49	65.49	54.47
	Wynne	83.43	81.82	78.14	72.29	55.10
Fish 3	Obtained	100.00	100.00	100.00	100.00	100.00
	Siemann-Delius	100.00	100.00	100.00	99.97	41.35
	Wynne	100.00	100.00	100.00	100.00	100.00
Fish 4	Obtained	100.00	100.00	100.00	100.00	100.00
	Siemann-Delius	100.00	99..99	100.00	99.99	27.08
	Wynne	99.99	98.91	99.97	99.69	43.20
