# Supplementary material for: Transitive inference in cleaner wrasses (Labroides dimidiatus)
Source: PLoS One. 2020 Aug 18;15(8):e0237817. doi: 10.1371/journal.pone.0237817 (PMC7433877; doi:10.1371/journal.pone.0237817)
Supplement: Attachment — Submitted filename: Response toward Reviewers.docx [file pone.0237817.s009.docx]

Response to reviewer #1

Comment 1

*In their manuscript, "Transitive inference in cleaner wrasses (Labroides dimidiatus)" authors Hotta, Ueno, Hataji, Kuroshima, Fujita, and Kohda describe the results of a transitive inference procedure conducted using cleaner wrasses. They assert (correctly, so far as I am aware) that this is the first demonstration of TI in fish that does not depend on a comparison of social rank.
For the most part, the experiment that the authors have conducted what in the abstract appears to be a very standard training procedure for a TI experiment. Their main innovation is to describe how this can be done in fish using stimuli that are more abstract in character than has been demonstrated previously, and they have included a sufficient level of detail that an interested party could replicate the experiment without difficulty. This on its own is a substantial contribution, as it provides a template for implementing this cognitive probe in a much wider range of species. The analysis and interpretation of the data are clear and convincing. On its experimental merits alone, I think this manuscript is suitable for publication.*

Response 1

We wish to express our appreciation to Reviewer #1 for his (or her) insightful comments, which have helped us significantly improve the paper. As pointed out, we added the information on simulations and the discussion the difference in previous study in other fish.

Comment 2
*My main advice for improving the manuscript relates to the theoretical calculation of associative strength using value transfer models. The reader is told that these models were fit "with or without value transfer modifications" but any further details about this implementation are omitted. Since the analyses were conducted in R, I think it would be beneficial to future work in this area for the authors to add an annotated R script to their supplemental information that a reader could download and run to replicate the process of fitting parameters using these models, in order to confirm the predicted behavior at test that is reported in Figure 3. Since the comparison of behavior to that of associative models is central to the claims made in the paper, the authors would do the field a favor by showing their work at this stage.*

Response 2

Thank you for your advices. All simulations were performed by MATLAB and we presented the script as supplemental file (Zip file). We added this sentence in L. 233-234.

Comment 3
*I also think the authors should devote at least a paragraph of their discussion to comparing this study to other studies that have examined TI in fish. The work by Grosenick and colleagues (2007) was surprising to at least a few comparative psychologists. That study's reliance on dominance hierarchies set the stage for subsequent TI procedures, but also carried with it the implied limitation that this might be a specialized form of cognition, limited to the social arena. The authors should revisit more explicitly the claim they make in the introduction that the current study demonstrates TI in a more abstract way than has been accomplished in fish to date, which in turn should strengthen the reader's confidence that past reports of TI in fish are not merely quirks of social cognition, but may indeed reflect a more fundamental inferential capacity.*

Response 3

Thank you for your suggestion. We agree that our study strengthen previous researches on transitive inference in fish. Previous studies (e.g. Grosenick et al. 2007) surely revealed that some fish have a capable of TI in social context, but it conducted with the implicated limitation that it may be a specialized form of cognition in dominance relationship. However, we demonstrated in a similar way such as mammals and birds in more abstract context. Thus, our results indicated that the TI ability found in previous studies are not merely social cognition, but also reflect a more fundamental inference capability in fish.

Like this, we added a paragraph for discussion in L. 344-357.
